# Usefulness of prolactin levels in predicting the etiology of hyperprolactinemia in a cohort of 770 patients

**DOI:** 10.20945/2359-4292-2023-0391

**Published:** 2024-09-11

**Authors:** Lucio Vilar, Clarice Freitas Vilar, Ruy Lyra, Luciano Albuquerque, Ana Carolina Thé Garrido, Patrícia Sampaio Gadelha, Erik Trovão Diniz, Marcos Almeida, Lucia Helena Cordeiro, Erico Higino de Carvalho, Ana Teresa Bezerra de Melo, Karoline Matias Medeiros, Gabriel Rodrigues de Assis Ferreira, José Coelho Mororó, Daniela Zago Ximenes, Camila Ribeiro Coutinho Madruga, Rosália de Oliveira Nunes, Yanna Queiroz Pereira de Sá, Luciana Ansaneli Naves

**Affiliations:** 1 Divisão de Endocrinologia Hospital das Clínicas Universidade Federal de Pernambuco Recife PE Brasil Divisão de Endocrinologia, Hospital das Clínicas, Universidade Federal de Pernambuco, Recife, PE, Brasil; 2 Centro de Pesquisas Endócrinas de Pernambuco Recife PE Brasil Centro de Pesquisas Endócrinas de Pernambuco, Recife, PE, Brasil; 3 Divisão de Endocrinologia Hospital Universitário de Brasília Brasília DF Brasil Divisão de Endocrinologia, Hospital Universitário de Brasília, Brasília, DF, Brasil

**Keywords:** Prolactin, hyperprolactinemia, microprolactinoma, prolactin-secreting pituitary adenoma, macroprolactinoma

## Abstract

**Objective:**

Determining the etiology of hyperprolactinemia is fundamental for selecting the most appropriate treatment strategy. The aim of this study was to evaluate the usefulness and accuracy of prolactin levels in predicting the etiology of nonphysiological hyperprolactinemia.

**Subjects and methods:**

In this retrospective study, we reviewed medical records of patients with nonphysiological hyperprolactinemia seen at two neuroendocrine reference centers located in Recife, Brazil, from January 2000 to December 2019.

**Results:**

The study included 770 patients aged 12-73 years (65% female). The three most frequent etiologies of hyperprolactinemia were prolactinomas (n = 263; 34.2%), drug-induced hyperprolactinemia (n = 160; 20.8%), and macroprolactinemia (n = 120; 15.6%). The highest mean prolactin levels were observed in cases of prolactinomas and idiopathic hyperprolactinemia. Most patients with hyperprolactinemia due to other etiologies had prolactin levels < 100 ng/mL, but these levels were also found in 16.5% of patients with microproplactinomas and in 20% of those with idiopathic hyperprolactinemia. Likewise, prolactin levels largely overlapped among patients with microprolactinomas, macroprolactinemia, and drug-induced hyperprolactinemia. Notably, prolactin levels > 250 ng/mL enabled a clear distinction between the etiologies of macroprolactinoma and nonfunctioning pituitary adenoma. Moreover, prolactin levels > 500 ng/mL were highly suggestive of macroprolactinomas, although they were also found in very few patients (<2%) with microprolactinomas or drug-induced hyperprolactinemia.

**Conclusion:**

Despite considerable overlap in prolactin levels among the different etiologies of hyperprolactinemia, values > 250 ng/mL allowed a clear distinction between macroprolactinomas and nonfunctioning pituitary adenomas. Furthermore, prolactin levels > 500 ng/mL were almost exclusively found in patients with prolactinomas.

## INTRODUCTION

Hyperprolactinemia is the most common abnormality of the hypothalamic-pituitary axis, leading to hypogonadotropic hypogonadism and infertility in both sexes (1). Causes of hyperprolactinemia can be divided into physiological, pathological, and pharmacological (2). Drugs and prolactinomas are usually considered the main causes of nonphysiological hyperprolactinemia (3,4).

The human prolactin has three major circulating molecular isoforms (5). The monomeric prolactin (little prolactin) has a molecular mass of 23 kDa and represents 80%-95% of the total prolactin in healthy subjects and in those with prolactinomas (5,6). Dimeric prolactin (big prolactin) has 45-60 kDa and makes up less than 10%, whereas polymeric prolactin (molecular mass > 150 kDa), also known as macroprolactin or big-big prolactin, accounts for less than 1% of the total prolactin (5-7). When more than 60% of circulating prolactin is made up of macroprolactin, this condition is termed macroprolactinemia (7-11).

According to many studies, the highest prolactin levels are found in patients with prolactinomas, whereas prolactin levels < 100 ng/mL are typically seen in individuals with macroprolactinemia, systemic diseases, drug-induced hyperprolactinemia, and pseudoprolactinomas (1,4,5,12), mainly represented by nonfunctioning pituitary adenomas (13). However, in the Brazilian Multicenter Study on Hyperprolactinemia, there was considerable overlap in prolactin levels among the different etiologies of nonphysiological hyperprolactinemia (13). Similar findings were reported in another Brazilian study comparing patients with monomeric hyperprolactinemia *versus* macroprolactinemia (14).

The main aim of this study was to assess the usefulness and accuracy of the magnitude of prolactin level in predicting the etiology of nonphysiological hyperprolactinemia.

## SUBJECTS AND METHODS

### Patients and study design

In this retrospective study, we reviewed the medical records of patients with nonphysiological hyperprolactinemia seen from January 2000 to December 2019 in two neuroendocrine reference centers in Recife, Brazil, *i.e.*, the Division of Endocrinology of Hospital das Clínicas at Pernambuco Federal University, and Pernambuco Center for Diabetes & Endocrinology.

A detailed form was filled out containing clinical, biochemical, and neuroradiological data, as well as the etiology of hyperprolactinemia and the chosen therapeutic option(s).

### Hormonal and biochemical parameters

Prolactin levels were measured using commercially available chemiluminescence immunoassays. Hyperprolactinemia was defined as a prolactin level above the normal range. The laboratory evaluation for the etiology of hyperprolactinemia included the measurement of other hormones (b-hCG, TSH, free T_4_, and IGF-1 levels), the evaluation of renal and liver functions, as well as the screening for macroprolactin. The latter was done through the polyethylene glycol (PEG) precipitation method. In agreement with other studies (7,9,10,15), prolactin recoveries of < 40% and > 60% after PEG precipitation were used as the diagnostic criteria for macroprolactinemia and monomeric hyperprolactinemia, respectively. Patients with prolactin recovery < 40% but with the nonprecipitated component elevated above normal range were also included in the monomeric hyperprolactinemia group.

### Pituitary imaging

Pituitary magnetic resonance imaging (MRI) studies were performed in all patients with a likely pituitary mass, using a 1.5-Tesla unit with T1- and T2-weighted images before and after the administration of gadolinium-based contrast. Pituitary adenomas were classified according to size as microadenomas (<1 cm), macroadenomas (≥1 cm), or giant adenomas (>4 cm) (16,17).

### Statistical analysis

The chi-square test or Fisher’s exact test (when necessary) was used in the analysis of qualitative variables. P values < 0.05 were considered statistically significant. Results are expressed as percentages and mean ± standard deviation values unless otherwise indicated. The statistical analysis was performed using IBM SPSS, Version 20.0 (IBM Corp., Armonk, NY, USA).

### Ethical aspects

The study was approved by the Ethics and Human Research Committee of Health at Olinda Medical School (CAAE 76499823.3.0000.8033). All patients provided informed consent for the inclusion of their clinical and laboratory data in the present study.

## RESULTS

### Etiology of hyperprolactinemia

A total of 770 patients were enrolled in this retrospective study. The patients were subdivided according to the etiology of the hyperprolactinemia as follows: prolactinomas, 34.1% (n = 263, including 121 microprolactinomas and 142 macroprolactinomas); drug-induced hyperprolactinemia, 20.8% (n = 160); macroprolactinemia, 15.6% (n = 120); primary hypothyroidism, 9.5% (n = 73); nonfunctioning pituitary adenomas, 9.1% (n = 70); idiopathic hyperprolactinemia, 5.2% (n = 40); acromegaly, 3.9% (n = 30); and other etiologies 1.8% (n = 14) (Table 1). The latter group included patients with hypophysitis (n = 4), craniopharyngiomas (n = 3), empty sella (n = 2), dysgerminomas (n = 2), neurosarcoidosis (n = 1), meningioma (n = 1), and chordoma (n = 1).

**Table 1:** Patients characteristics and clinical features according to the etiology of hyperprolactinemia

Etiology of hyperprolactinemia	Sex*	Age, years**	Galactorrhea only***	Menstrual disorders only (female patients)	Menstrual disorders + galactorrhea (female patients)	Hypogonadism symptoms (male patients)	No symptoms***
Macroprolactinomas (n = 142)	82/60	32.3 ± 9.3 (12-55)	9.7 (F) 15 (M)	36.6	43.9	90.0	6.1 (F) 10 (M)
Microprolactinomas (n = 121)	90/31	32.4 ± 12.6 (13-58)	13.5 (F) 12.9 (M)	29.7	46.8	87.1	12.2 (F) 12.9 (M)
Drug-induced (n = 160)	90/70	39.1 ± 10.7 (18-64)	20 (F) 14.3 (M)	30.0	30.0	55.7	20 (F) 30 (M)
Macroprolactinemia (n = 120)	100/20	40.6 ± 9.8 (19-65)	13 (F) 0 (M)	26.0	3.0	50.0	58 (F) 50 (M)
Nonfunctioning pituitary adenomas (n = 70)	43/27	43.9 ± 15.2 (21-73)	12.8 (F) 7.4 (M)	34.9	22.2	77.8	30.1 (F) 14.8 (M)
Hypothyroidism (n = 73)	50/23	42.7 ± 9.7 (25-62)	26.6 (F) 0 (M)	35.6	0.0	50.0	40 (F) 50 (M)
Idiopathic (n = 40)	35/5	37.5 ± 7.6 (28-49)	15 (F) 0 (M)	32.0	45.0	80.0	10 (F) 20 (M)
Acromegaly (n = 30)	17/13	41.9 ± 7.3 (26-52)	13 (F) 7.7 (M)	23.3	21.7	84.6	8.7 (F) 15.4 (M)

* Shown as number of female patients/number of male patients. ** Shown as mean ± standard deviation (range). *** Shown as percentages of female patients/percentage of male patients. All other values are shown as percentages of patients.

Screening for macroprolactin was done in 60 patients who were previously diagnosed with idiopathic hyperprolactinemia. The results confirmed macroprolactinemia in 17 (28.3%) of these patients. Five patients with microprolactinomas (4.1%) had concomitant primary hypothyroidism. Likewise, 5 patients with microprolactinomas had prolactin recovery < 40% in the PEG precipitation test, a finding indicative of macroprolactinemia but, in these cases, the value of monomeric prolactin was above the normal range.

**Figure 1:**
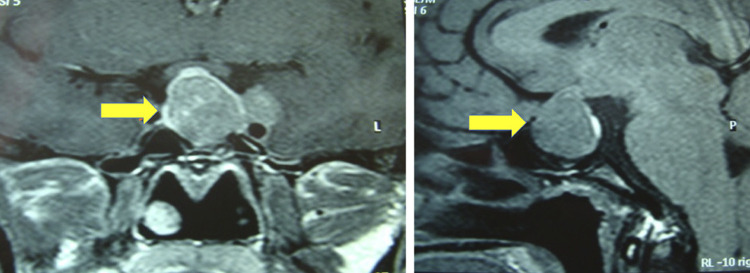
Coronal (left) and sagittal (right) magnetic resonance imaging of the pituitary in a 28-year-old female patient with a prolactin level of 343 ng/mL and a 2.8 cm macroadenoma (arrows) that was initially labeled a cabergoline-resistant prolactinoma. After polyethylene glycol (PEG) precipitation, the prolactin level decreased to 27.1 ng/mL (normal range < 29 ng/mL). The patient received a final diagnosis of gonadotroph pituitary adenoma associated with macroprolactinemia.

The screening for macroprolactin also enabled us to differentiate nonfunctioning adenomas from prolactinomas in some cases. For example, a patient with a 2.8 cm macroadenoma and an initial prolactin level of 343 ng/mL was referred to us with a diagnosis of cabergoline-resistant macroprolactinoma (Figure 1). Monomeric prolactin concentration after PEG precipitation test was normal (27.1 ng/mL; normal range, up to 29 ng/mL). The patient was submitted to transsphenoidal surgery, and the final diagnosis was gonadotroph pituitary adenoma associated with macroprolactinemia.

Concerning drug-induced hyperprolactinemia, most cases (n = 110; 68.7%) were related to the use of antipsychotics and antidepressants, in monotherapy or in combination. The remaining cases were related to the use of estrogens (n = 15) or prokinetics (n = 35), particularly domperidone. Among antipsychotics, the most frequently involved were haloperidol, phenothiazines, and risperidone while antidepressants were mainly represented by selective serotonin reuptake inhibitors.

### Patients’ characteristics and clinical features

The mean age of the patients was 43.28 ± 15.30 years (range, 12-73 years; median, 43 years). There was a predominance of female patients (n = 507; 65.8%) regardless of the etiology of the hyperprolactinemia (Table 1).

**Figure 2:**
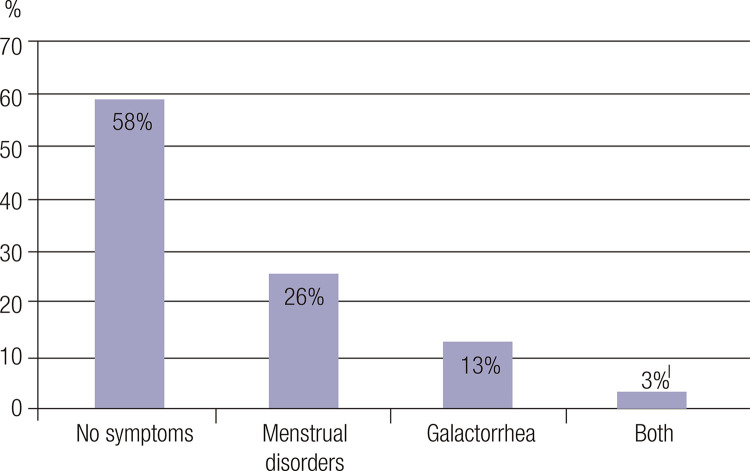
Clinical features of 100 women with macroprolactinemia.

Among female patients with prolactinomas or idiopathic hyperprolactinemia, 77%-83.5% reported menstrual disorders (oligomenorrhea or amenorrhea) with or without the presence of galactorrhea. Clinical manifestations indicative of hyperprolactinemia were found in 50% of male patients and in 42 out of 100 female patients (42%) with macroprolactinemia. In the latter group, 26% had only menstrual disorders and 13% had only galactorrhea, while approximately 3% had both (Figure 2). Among the 20 male patients, 5 (25%) had erectile dysfunction, 3 (15%) had decreased libido, 1 (5%) had both these manifestations, and 11 (55%) were asymptomatic. In patients with prolactinomas or idiopathic hyperprolactinemia, the mean rate of menstrual disorders combined with galactorrhea ranged from 44% to 47% (Table 1). The rate of symptoms in patients with macroprolactinemia was similar in cases with prolactin levels < 100 ng/mL or ≥ 100 ng/mL (51.9% *versus* 48.1%, respectively, p = 0.910). By contrast, among subjects with microprolactinomas, only 12.5% were asymptomatic, with the absence of symptoms prevailing in patients with prolactin < 100 ng/mL.

### Magnetic resonance imaging findings

In patients with microprolactinomas, the adenoma size ranged from 0.5 to 0.9 cm. Among the 142 cases of macroprolactinomas, there were 14 giant adenomas (4.6-9.1 cm). All patients with nonfunctioning pituitary adenomas had macroadenomas (1.6-5.2 cm). Pituitary abnormalities on MRI were also depicted in 28 patients (23.3%) with macroprolactinemia: microadenomas in 16 (13.3%), empty sella in 8 (6.7%), and macroadenomas in 4 (3.3%) (Figure 1).

### Prolactin levels

As shown in Table 2 and commented below, the highest prolactin levels were observed in patients with macroprolactinomas and the lowest in individuals with primary hypothyroidism. However, there was considerable overlap in prolactin values regardless of the etiology of the hyperprolactinemia. Table 3 details the distribution of patients according to the etiology of hyperprolactinemia and prolactin levels.

**Table 2:** Prolactin levels according to the etiology of the hyperprolactinemia

Etiology	Number (%) of patients	Prolactin levels* (ng/mL)
Macroprolactinomas	142 (18.4)	1,230.17 ± 2,395.02 (109-22,600)
Microprolactinomas	121 (15.7)	196.04 ± 97.86 (51-525)
Macroprolactinemia	120 (15.6)	88.70 ± 45.60 (40-490)
Drug-induced	160 (20.8)	86.97 ± 112.90 (37-720)
Primary hypothyroidism	73 (9.5)	81.33 ± 49.34 (34-253)
Nonfunctioning pituitary adenomas	70 (9.1)	86.97 ± 45.14 (39-250)
Idiopathic	40 (5.2)	157.50 ± 67.45 (52-317)
Acromegaly	30 (3.9)	104.17 ± 65.39 (37-310)
Other etiologies**	14 (1.8)	113.76 ± 61.87 (46-226)

* Shown as mean ± standard deviation (range). ** Includes hypophysitis (n = 4), craniopharyngiomas (n = 3), empty sella (n = 2), dysgerminomas (n = 2), neurosarcoidosis (n = 1), meningioma (n = 1), and chordoma (n = 1).

**Table 3:** Distribution of prolactin levels according to the etiology of the hyperprolactinemia

Etiology of hyperprolactinemia (number of patients)	Prolactin levels (ng/mL)					
	Up to 49	50-100	101-200	201-250	251-500	>500
Macroprolactinomas (n = 142)	0.0	0.0	4.9	9.1	35.2	50.8
Microprolactinomas (n = 121)	0.0	16.5	41.3	24.8	15.7	1.7
Drug-induced (n = 160)	15.6	48.8	18.7	10.0	5.0	1.9
Macroprolactinemia (n = 120)	16.7	48.3	16.7	11.6	6.7	0.0
Nonfunctioning pituitary adenomas (n = 70)	27.1	51.5	17.1	4.3	0.0	0.0
Primary hypothyroidism (n = 73)	20.5	43.8	24.7	9.6	1.4	0.0
Idiopathic (n = 40)	0.0	20.0	45.0	20.0	15.0	0.0
Acromegaly (n = 30)	3.3	63.4	20.0	10.0	3.3	0.0
Other etiologies (n = 14)*	14.3	42.8	28.6	14.3	0.0	0.0

* Includes hypophysitis (n = 4), craniopharyngiomas (n = 3), empty sella (n = 2), dysgerminomas (n = 2), neurosarcoidosis (n = 1), meningioma (n = 1), and chordoma (n = 1).

### Macroprolactinomas

**Figure 3:**
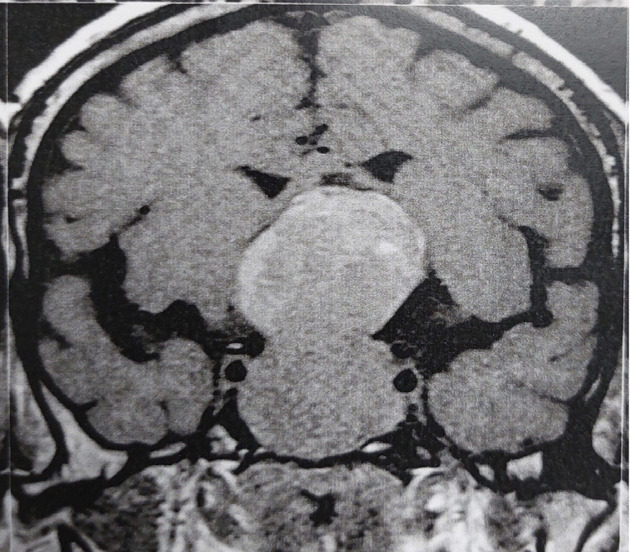
Coronal magnetic resonance imaging of the pituitary showing a giant prolactinoma (6.4 cm) in a patient with falsely low serum prolactin level (91 ng/mL) due to the high-dose hook effect. The prolactin level increased to 22,600 ng/mL after a 1:100 dilution of the serum sample.

The prolactin levels in patients with macroprolactinomas (n = 142) ranged from 109 to 22,600 ng/mL (mean, 1,230.17 ± 2,395.02 ng/mL; median, 550 ng/mL). The levels were distributed as follows among the patients (represented in %): < 100 ng/mL, 0%; 100-250 ng/mL, 14.1%; 251-500 ng/mL, 35.2%; > 500 ng/mL, 50.8%. Among the 14 patients with giant prolactinomas (> 4 cm), prolactin values ranged between 3,700 and 22,600 ng/mL. In 2 of them, the initial prolactin was < 100 ng/mL due to the high-dose hook effect, which was unmasked by repeating prolactin measurement after serum dilutions (Figure 3).

### Microprolactinomas

The prolactin levels in patients with microprolactinomas (n = 121) ranged from 51 to 525 ng/mL (mean, 196.04 ± 97.86 ng/mL; median, 181 ng/mL). The levels were distributed as follows among the patients (%): <100 ng/mL, 16.5%; 100-250 ng/mL, 66.1%; 251-500 ng/mL, 15.7%; >500 ng/mL, 1.7%.

### Drug-induced hyperprolactinemia

The prolactin levels in patients with drug-induced hyperprolactinemia (n = 160) ranged from 37 to 720 ng/mL (mean, 86.97 ± 112.90 ng/mL; median, 85 ng/mL). The levels were distributed as follows among the patients (%): < 100 ng/mL, 64.3%; 101-250 ng/mL, 28.7%; 251-500 ng/mL, 5%. Of 3 patients (1.9%) with prolactin levels > 500 ng/mL, 2 were taking domperidone (505 and 720 ng/mL) and 1 was taking risperidone (514 ng/mL).

### Macroprolactinemia

The prolactin levels in patients with macroprolactinemia (n = 120) ranged from 40 to 490 ng/mL (mean, 88.70 ± 45.60 ng/mL; median, 77 ng/mL). The levels were distributed as follows among the patients (%): <100 ng/mL, 65%; 100-250 ng/mL, 28.3%; 251-500 ng/mL, 6.7%; >500 ng/mL, 0%. The mean prolactin levels in patients with macroprolactinemia were lower than those observed in patients with microprolactinomas (p < 0.01), idiopathic macroprolactinemia (p < 0.01), or macroprolactinomas (p < 0.001). However, considerable overlap was observed, as shown in Table 2.

### Primary hypothyroidism

The prolactin levels in patients with hyperprolactinemia due to primary hypothyroidism (n = 73) ranged from 34 to 253 ng/mL (mean, 81.33 ± 49.34 ng/mL; median, 64 ng/mL). The levels were distributed as follows among the patients (%): <100 ng/mL, 24.6%; 101-250 ng/mL, 74.0%; 251-500 ng/mL, 1.4%; >500 ng/mL, 0%.

### Nonfunctioning pituitary adenomas

The prolactin levels in patients with hyperprolactinemia and nonfunctioning pituitary adenomas (n = 70) ranged from 39 to 250 ng/mL (mean, 86.97 ± 45.14 ng/mL; median, 77 ng/mL). The levels were distributed as follows among the patients (%): <100 ng/mL, 78.6%; 101-250 ng/mL, 21.4%; >250 ng/mL, 0%.

### Idiopathic hyperprolactinemia

The prolactin levels in patients with idiopathic hyperprolactinemia (n = 40) ranged from 52 to 317 ng/mL (mean, 157.50 ± 67.45 ng/mL; median, 161.45 ng/mL). The levels were distributed as follows among the patients (%): <100 ng/mL, 20%; 101-250 ng/mL, 65%; 251-500 ng/mL, 15%; >500 ng/mL, 0%.

### Acromegaly

The prolactin levels in patients with hyperprolactinemia due to acromegaly (n = 30) ranged from 37 to 310 ng/mL (mean, 104.17 ± 65.39 ng/mL; median, 75.5 ng/mL). The levels were distributed as follows among the patients (%): <100 ng/mL, 66.8%; 101-250 ng/mL, 29.9%; 251-500 ng/mL, 3.3%; >500 ng/mL, 0%.

### Other etiologies

The prolactin levels in patients with hyperprolactinemia due to other etiologies (n = 14) ranged from 46 to 226 ng/mL (mean, 113.76 ± 61.87 ng/mL; median, 86.5 ng/mL). The levels were distributed as follows: <100 ng/mL, 57.1%; 101-250 ng/mL, 42.9%; >250 ng/mL, 0%.

## DISCUSSION

In this retrospective study of 770 patients with hyperprolactinemia, the three most frequent causes of hyperprolactinemia were prolactinomas, drug-induced hyperprolactinemia, and macroprolactinemia. Similar results were found in the Brazilian Multicenter Study on Hyperprolactinemia, which involved 1,234 patients and was published in 2008 (13).

Defining the etiology of hyperprolactinemia is fundamental for choosing the appropriate treatment (12,18). Some controversy still remains regarding the relevance of the magnitude of prolactin level elevation in predicting the etiology of hyperprolactinemia. This is particularly true in the differentiation between macroprolactinomas and pseudoprolactinomas, mostly represented by nonfunctioning pituitary adenomas, as they require distinct treatments (5,18-20). In cases of nonfunctioning pituitary adenomas, hyperprolactinemia is usually much milder (most cases have prolactin levels < 100 ng/mL) as it is thought to mainly result from stalk compression that hampers the suppression of lactotrophs by dopamine, and not from prolactin hypersecretion, as it occurs with prolactinomas (1,2,18-20). In the current study, 75% of patients with nonfunctioning pituitary adenomas confirmed by immunohistochemistry had prolactin levels < 100 ng/dL, with the remaining values ranging from 100 to 250 ng/mL. In a British study (n = 226), prolactin levels > 2,000 mU/L (94 ng/mL) were observed only in 3 cases of nonfunctioning pituitary adenomas (1.3%) and the maximum values were 3,257 mU/L (153 ng/mL) and 2,565 mU/L (120 ng/mL) in patients taking and not taking drugs capable of increasing prolactin levels, respectively (21). Similar results were reported by Behan and cols. (22). In a more recent study, involving 214 patients with nonfunctioning pituitary adenomas, preoperative hyperprolactinemia was found in 93 cases (43.5%), and median serum prolactin concentration was 34.68 ng/mL (range, 0.23-213.90 ng/mL) (19). By contrast, in the Brazilian Multicenter Study on Hyperprolactinemia, the highest prolactin level was 490 ng/mL (13). However, in this particular case, the tumor was not evaluated by immunohistochemistry and could actually be a macroprolactinoma.

In patients with prolactinomas, circulating prolactin levels usually parallel tumor size and the amount of tumor cells (1-4). Hence, the highest prolactin levels are found in patients with larger macroprolactinomas, as shown in the present study and in many others (1,5,16,23,24). Some authors have recently described that young patients with invasive macroprolactinomas may present higher prolactin levels when these tumors are resistant to dopamine agonists, which may be associated with genetic causes like multiple endocrine neoplasia (MEN) 1 and pathogenic germline variants in the *AIP* gene (AIPvar) (25).

Artificially normal or mildly elevated prolactin values can be seen in cases of giant prolactinomas due to the so-called high-dose hook effect (1,3,26). This phenomenon can be unmasked by repeating prolactin measurement after serum dilutions (26). Mild prolactin elevation may also occur in cases of cystic or hemorrhagic adenomas (1,3,18,27). Thus, once the possibility of the hook effect is excluded, a prolactin value < 100 ng/mL in a patient harboring a solid pituitary macroadenoma virtually excludes a macroprolactinoma and is highly indicative of a nonfunctioning pituitary adenoma (1,3). By contrast, patients with microprolactinomas may have prolactin levels < 100 ng/mL, as seen in 16.5% of the patients in the present study. In the Brazilian Multicenter Study on Hyperprolactinemia, this proportion was 25% (13).

Notably, the high-dose hook effect is currently rare with the new immunoassays for prolactin measurement. For example, the Roche Cobas Prolactin II assay shows no high-dose hook effect at prolactin concentrations up to approximately 12,690 ng/mL (28). In the study by Raverot and cols. (29), serum from a patient with a giant macroprolactinoma was assayed using all the available prolactin assays in France in 2020, both on native serum and after dilution. Fourteen assay kits were studied by 16 laboratories; all were two-site immunometric assays, mostly using one step. The results obtained after dilution varied from 17,900 µg/L to 86,900 µg/L depending on the assay used. Only one tested assay was sensitive to the high-dose hook effect leading to a falsely lower prolactin concentration when measuring native serum (150 µg/L compared with 17,900 µg/L after dilution) (29).

Although macroprolactinemia is the third most frequent cause of hyperprolactinemia (30), there is still no consensus on routine screening for macroprolactin in patients with elevated prolactin levels (3,4,31). The 2011 Endocrine Society guidelines recommended routine screening for macroprolactin only in asymptomatic patients with hyperprolactinemia (31). According to the recommendations of the Department of Neuroendocrinology of the Brazilian Society of Endocrinology and Metabolism (SBEM), indications for macroprolactin screening should include asymptomatic patients, individuals with idiopathic hyperprolactinemia, and those without an obvious etiology for the hyperprolactinemia (3). Due to the low macroprolactin biological activity (11), most patients with macroprolactinemia lack clinical manifestations related to hyperprolactinemia (7-10). However, the presence of these manifestations or neuroradiological abnormalities does not exclude the diagnosis of macroprolactinemia, as shown in some series and in the present study. For instance, in three European series involving 212 patients with macroprolactinemia, 4.9%-33% had infertility, 12.3%-59% had menstrual disorders, and 22%-46% had galactorrhea (8,9,32). Among 100 female patients with macroprolactinemia in the present study, 42% had symptoms including menstrual disorders and galactorrhea. Likewise, 26% had pituitary abnormalities ranging from empty sella to macroadenoma. The presence of symptoms in cases of macroprolactinemia is often attributed to the co-occurrence of other disorders (1,4,8,10,32). Some private laboratories in Brazil routinely screen for macroprolactin in patients with prolactin levels above the normal range.

Another interesting finding of our study was the wide variability in prolactin levels in patients with macroprolactinemia or drug-induced hyperprolactinemia. In fact, although most patients with these conditions have prolactin levels < 100 ng/mL, values overlapping those seen in cases of prolactinomas were also observed. In particular, 3 patients with drug-induced hyperprolactinemia had prolactin concentrations above 500 ng/mL, reaching 720 ng/mL in a patient who was taking domperidone and also harbored a 0.5 cm nonfunctioning pituitary microadenoma. At 20 days after domperidone withdrawal, prolactin was normal. In patients with macroprolactinemia, the maximum prolactin value observed was 490 ng/mL.

Among our patients with apparent idiopathic hyperprolactinemia, macroprolactin screening led to a change in diagnosis to macroprolactinemia in one-third of cases, half of which had been improperly treated with cabergoline. Similar findings have been reported in other studies (31). The screening also enabled to differentiate nonfunctioning microadenomas and macroadenomas from prolactinomas. Notably, the screening for macroprolactin enabled us to define the diagnosis of a gonadotroph pituitary adenoma in a patient with a 2.8 cm macroadenoma and an initial prolactin level of 343 ng/mL who was referred to us with a diagnosis of cabergoline-resistant macroprolactinoma. Thus, the findings of our study indicate that macroprolactin is worth screening in all patients with pituitary tumors and prolactin < 500 ng/mL, regardless of their clinical presentation.

In conclusion, despite considerable overlap in prolactin levels among different etiologies of hyperprolactinemia, values > 250 ng/mL allowed a clear distinction between macroprolactinomas and nonfunctioning pituitary adenomas. Furthermore, prolactin levels > 500 ng/mL were almost exclusively found in patients with prolactinomas but also in < 2% of the patients with microprolactinomas or drug-induced hyperprolactinemia.
